# Expression of the cancer stem cell markers ABCG2 and OCT-4 in right-sided colon cancer predicts recurrence and poor outcomes

**DOI:** 10.18632/oncotarget.15307

**Published:** 2017-02-14

**Authors:** Jun Hu, Jian Li, Xin Yue, Jiacang Wang, Jianzhong Liu, Lin Sun, Dalu Kong

**Affiliations:** ^1^ Department of Colorectal Cancer Surgery, National Clinical Research Center for Cancer, Key Laboratory of Cancer Prevention and Therapy of Tianjin, Tianjin's Clinical Research Center for Cancer, Tianjin Medical University Cancer Institute and Hospital, Tianjin 300060, P.R. China; ^2^ Department of Lymphoma, State Key Laboratory of Experimental Hematology, Institute of Hematology and Blood Disease Hospital, Chinese Academy of Medical Sciences and Peking Union Medical College, Tianjin 300020, P.R. China; ^3^ Department of Pathology, National Clinical Research Center for Cancer, Key Laboratory of Cancer Prevention and Therapy of Tianjin, Tianjin's Clinical Research Center for Cancer, Tianjin Medical University Cancer Institute and Hospital, Tianjin 300060, P.R. China

**Keywords:** stem cell, markers, abcg2, oct-4, prognosis

## Abstract

Right-sided colon cancer (RCC) has a poorer prognosis and a higher relapse rate than left-sided colon cancer (LCC). Like cancer stem cells (CSCs), RCC cells cannot be fully eradicated and are often involved in relapse or metastasis. Because CSCs may be linked with poor outcomes, CSC markers may have prognostic value in RCC. ATP-binding cassette sub-family G member 2 (ABCG2) and OCT-4 (also known as POU5F1) are among the most useful markers for CSC identification. We therefore examined the malignant behavior of ABCG2 and OCT-4 *in vitro* and *in vivo*, and their expression was assessed in pathology tissues obtained from clinicopathologically recurrent and non-recurrent cases. Our survey suggested associations between ABCG2 and OCT-4 expression and RCC clinicopathological variables. No correlations were detected between ABCG2 or OCT-4 expression and age, gender, tumor size, or tumor shape, but ABCG2 expression correlated with TNM stage, tumor differentiation, and lymphovascular invasion. Additionally, expression of both ABCG2 and OCT-4 correlated with RCC recurrence and poor outcomes.

## INTRODUCTION

Colon cancer is among the most common malignant diseases, and its morbidity increases annually [1]. The situation in China may be worse than the global situation [2, 3]. Recently, there have been numerous reports describing correlations between colon cancer prognosis and anatomical location [4] and confirming a significantly worse prognosis and higher risk of recurrence for right-sided colon cancer (RCC) than for left-sided colon cancer (LCC) [5, 6].

According to the cancer stem cell (CSC) theory, only a small number of cells in a tumor have the potential to self-renew and differentiate to maintain malignancy [7–9]. These CSCs are considered tumor-initiating cells (TICs) and have been identified in colorectal cancers and numerous other malignant tumors [10]. The presence of CSCs facilitates tumor relapse and tumor chemotherapy resistance, even when chemotherapies are applied in combination with targeted drugs. These characteristics are similar to the biological features of RCC, which demonstrates a high level of recurrence and a reduced survival rate compared with LCC. Unfortunately, few reports describing the relationship between RCC prognosis and CSCs, particularly CSC markers, are available. However, ATP-binding cassette sub-family G member 2 (ABCG2) and OCT-4 (also known as POU5F1 (POU family of transcription factors, class 5, factor 1)) are accepted CSC markers in numerous cancers and are linked with prognosis. ABCG2 is an ATP-binding cassette (ABC) efflux transporter that was recently accepted by the Food and Drug Administration (FDA) as a critical transporter involved in drug removal from the cell [11]. OCT-4 is a transcription factor that has been shown to play a significant role in tumorigenesis and embryogenesis and is associated with maintenance of stemness and cancer prognosis [12]. In this study, we investigated whether ABCG2 and OCT-4 expression are associated with the clinicopathological features of recurrent and non-recurrent cases of RCC.

## RESULTS

### Downregulating ABCG2 and OCT-4 inhibits CD133 expression, sphere formation, and tumorigenesis

**Figure 1:**
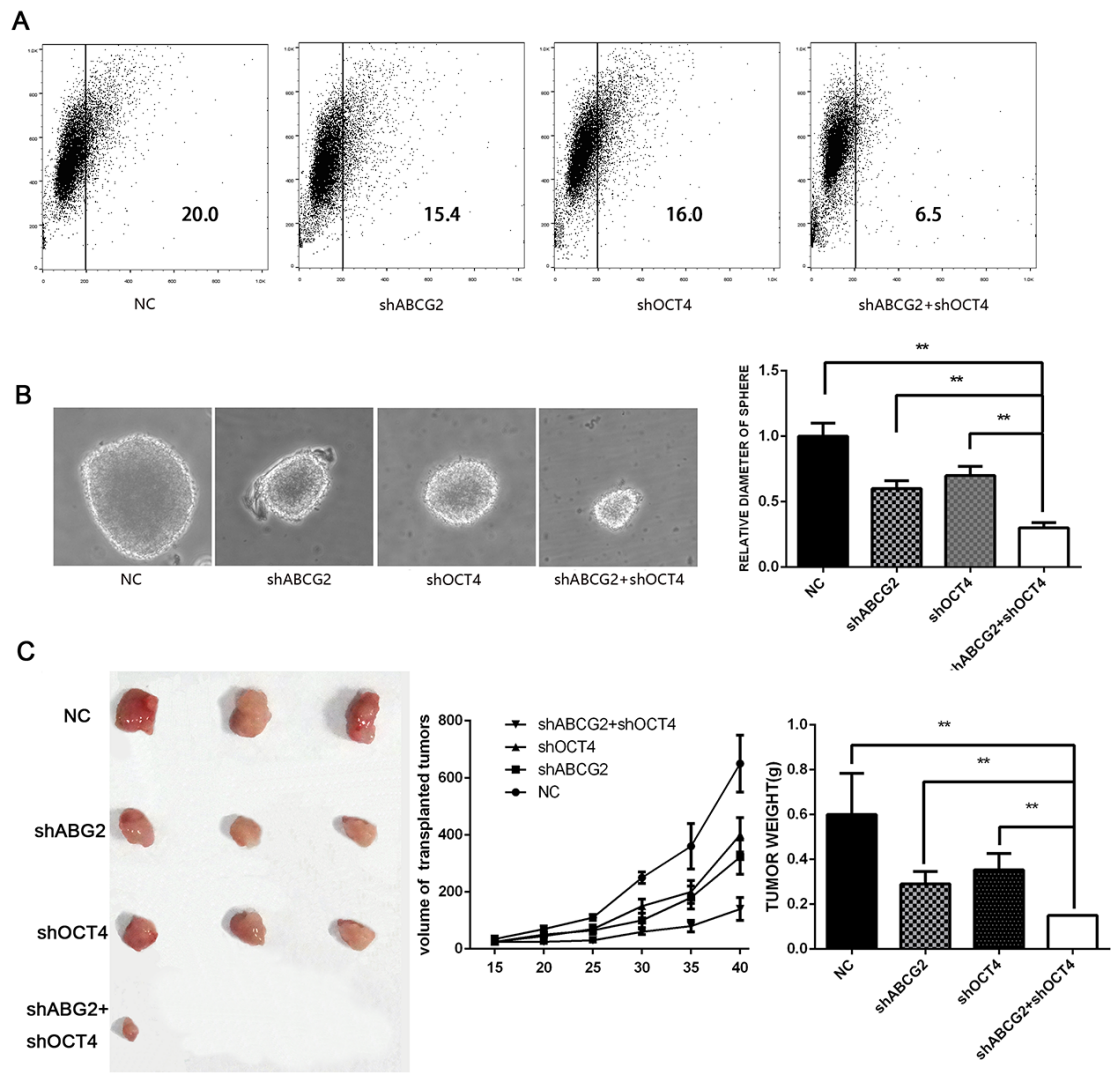
Downregulating ABCG2 and OCT-4 inhibits CD133 expression, sphere formation, and tumorigenic ability **A**. Proportion of CD133+ cells among transfected SW480 cells. At 48h after transfection with ABCG2 and OCT-4, the SW480 cells were resuspended, and CD133 was detected on the cell membrane. **B**. SW480 cells transfected with respective constructs were used for sphere formation and are shown at 14 days along with statistical analysis. **C**. Lentivirus-based sh-ABCG2-transfected and sh-OCT4-transfected SW480 cells were used for tumor initiation. The BALB/c-nude mice sacrificed six weeks after injection. All data were compared with the NC group.

Because ABCG2 and OCT-4 expression is significantly correlated with RCC recurrence, we suggest that ABCG2 and OCT-4 have a potential role in the development and maintenance of the stem cell–like properties of SW480 cells. Compared with control cells, sh-ABCG2 and sh-OCT-4 cells formed much smaller spheres after 7 days of culture (Figure 1A). Flow cytometry showed that sh-OCT-4 decreased the proportion of CD133+ cells (Figure 1B). To determine whether ABCG2 and OCT-4 are involved in SW480 cell tumorigenesis *in vivo*, we subcutaneously inoculated cells into the inguinal folds of nude mice. The tumors formed by sh-ABCG2 and sh-OCT-4 cells were visibly smaller than the vector control tumors (Figure 1C). Almost no tumor was formed by the simultaneously downregulated ABCG2 and OCT-4 cells.

### ABCG2 and OCT-4 were significantly correlated with RCC recurrence and poor outcomes

A total of 143 RCC patients were enrolled in the study. The patients’ ages ranged from 21 to 93 years (mean ± SD: 57.73 ± 12.32 years). The female-to-male ratio was 1.16:1. In total, 102 patients received chemotherapy, and 41 early-stage patients did not receive chemotherapy. Forty-seven patients experienced recurrence, and 96 patients did not experience recurrence after the operation. ABCG2 is localized to the membrane and cytoplasm, whereas OCT-4 is localized primarily in the cytoplasm. When the ABCG2 and OCT-4 expression status was compared with the clinicopathological variables, there were no correlations with age (*p*=0.994 *vs*. *p*=0.313), gender (*p*=0.104 *vs*. *p*=0.083), tumor size (*p*=0.073 *vs*. *p*=0.491), or tumor shape (*p*=0.485 *vs*. *p*=0.201). However, ABCG2 expression was significantly correlated with the TNM stage (*p*=0.000), the extent of differentiation (*p*=0.008), and lymphovascular invasion (*p*=0.002) of RCC. We did not identify statistical correlations between OCT-4 expression and the TNM stage (*p*=0.143), the extent of differentiation (*p*=0.055), or lymphovascular invasion (*p*=0.063). Our survey revealed significant differences between ABCG2 expression and RCC recurrence compared with non-recurrence (52.5% vs. 47.5%, *p*=0.002). Interestingly, OCT-4 expression was significantly correlated with RCC recurrence (42% vs. 58%, *p*=0.002) (Table 1) and was an independent indicator of RCC recurrence.

**Table 1:** Correlations between ABCG2 and Oct-4 expression and clinicopathologic characteristics in right-sided colon cancer

	No. of patients (%)	ABCG2 (%)	*P-*value	Oct-4 (%)	*P-*value
Age(years)	21-93				
Mean±SD	57.73±12.32		0.994		0.313
<60	74(51.7)	29(50.8)		23(46)	
≥60	69(48.3)	30(49.2)		27(54)	
Gender			0.104		0.083
Male	66(53.8)	32(50.2)		28(56)	
Female	77(43.2)	27(45.8)		22(44)	
Tumor size(mm)			0.073		0.491
≤50	10(51)	23(43.4)		28(56)	
51~99	60(42)	23(43.4)		20(40)	
≥100	73(7)	7(13.2)		2(4)	
pTNM stage			0.000		0.143
I	5(3.5)	1(1.7)		1(2)	
II	74(51.7)	20(33.9)		21(42)	
III	54(37.8)	34(57.6)		22(44)	
IV	10(7)	4(6.8)		6(12)	
Tumor differentiation			0.008		0.055
Well	15(10.5)	4(6.8)		2(4)	
Moderate	62(43.4)	13(22)		14(28)	
Poor	66(46.2)	42(71.2)		34(68)	
Lymphovascular invasion			0.002		0.063
Negative	75(52.4)	22(37.3)		22(44)	
Positive	68(47.6)	37(62.7)		28(56)	
Tumor shape			0.485		0.201
Massive	28(19.6)	9(15.3)		9(18)	
Ulcerative	81(56.6)	34(57.6)		33(66)	
Infiltrating	34(23.8)	16(27.1)		8(16)	
Recurrence			0.002		0.000
No	96(67.1)	31(52.5)		21(42)	
Yes	47(32.9)	28(47.5)		29(58)	

The rates of ABCG2 expression were 32.3% (31/96) and 59.6% (28/47) in the non-recurrent and recurrent cases, respectively (*p*=0.002). The rates of OCT-4 expression were 21.9% (21/96) and 61.7% (29/47) in the non-recurrent and recurrent cases, respectively (*p*=0.000).

**Figure 2:**
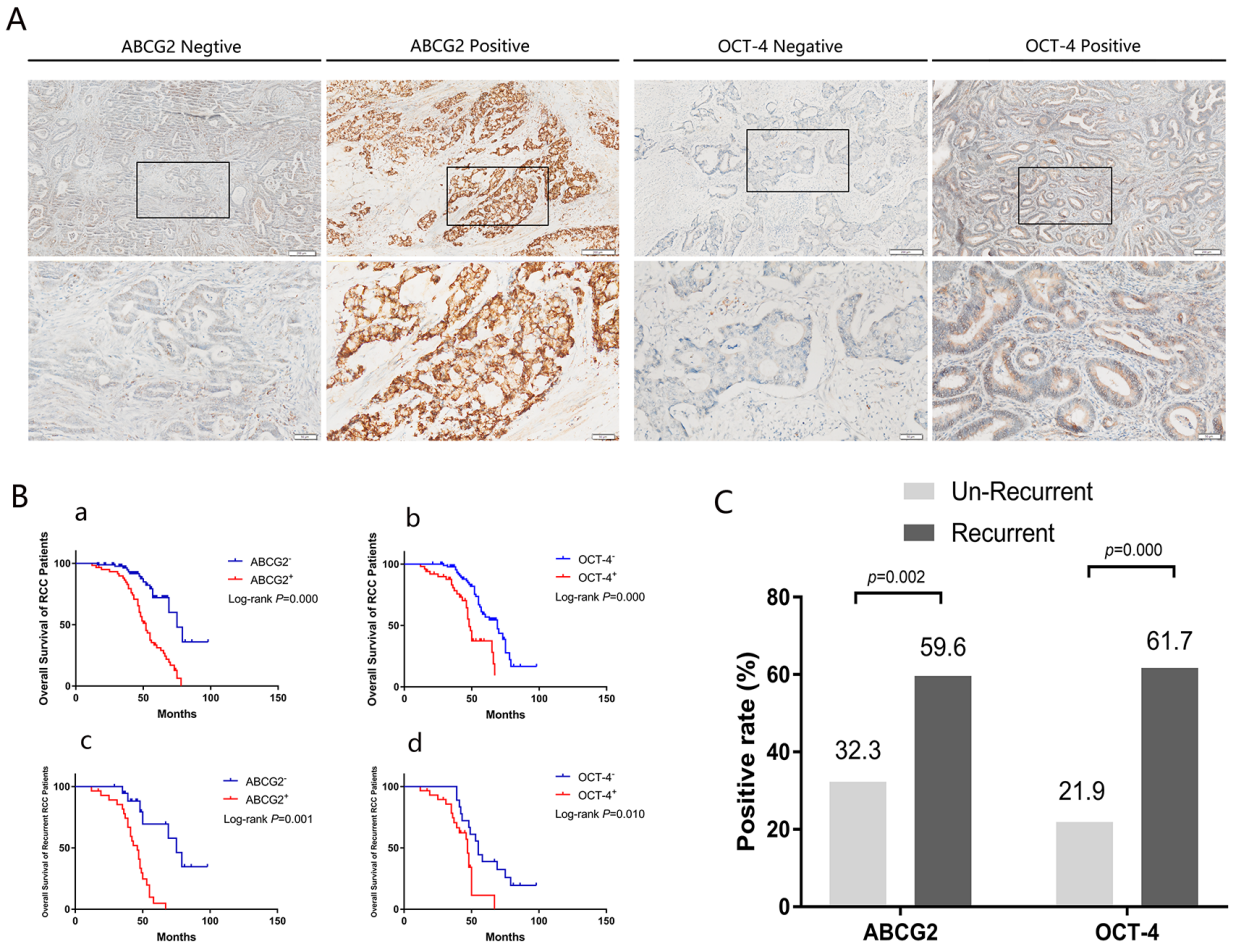
ABCG2 and OCT-4 were significantly correlated with RCC recurrence and predict a poor outcome **A**. Representative IHC staining of positive and negative expression of ABCG2 and OCT-4 is presented at 400× magnification and 100× magnification. Images in lower panels show higher magnifications of the areas boxed in upper panels. **B**. Kaplan-Meier analysis of overall survival in RCC with positive or negative expression of the stem cell marker ABCG2 and OCT-4. Both ACBG2 and OCT-4 were detected have poor prognosis in total groups (143 patients) and recurrent groups (47 patients). **a**. Overall survival curves of total groups (143 patients) with ABCG2 expression situation. **b**. Overall survival curves of total groups (143 patients) with OCT-4 expression situation. **c**. Overall survival curves of recurrent groups (47 patients) with ABCG2 expression situation. **d**. Overall survival curves of recurrent groups (47 patients) with OCT-4 expression situation. **C**. The positive expression rate of ABCG2 and OCT-4 in un-recurrent (96 patients) and recurrent groups (47 patients) of RCC.

The correlations between ABCG2 and OCT-4 expression and RCC prognosis were investigated by Kaplan-Meier analysis. The median survival periods of the ABCG2^−^ and ABCG2^+^ groups were 77.65 months and 51.32 months, respectively, whereas the median survival periods of the OCT4^−^ and OCT4^+^ groups were 68.11 months and 48.92 months. The five-year overall survival rates of the ABCG2^−^and ABCG2^+^ groups were 72.0% vs. 31.2%, respectively (*p*=0.000), whereas the five-year overall survival rates of the OCT-4^−^ and OCT-4^+^ groups were 56.9% vs. 37.5% (*p*=0.000). In the recurrent groups, the median survival periods of the ABCG2^−^ and ABCG2^+^ groups were 76.93 months and 45.41 months, respectively, whereas the median survival periods of the OCT-4^−^ and OCT-4^+^ groups were 56.67 months and 47.00 months. Furthermore, among the recurrent patients, the five-year overall survival rates of the ABCG2^−^ and ABCG2^+^ groups were 72% vs. 31.2%, respectively (*p*=0.000), whereas the five-year overall survival rates of the OCT-4^−^ and OCT-4^+^ groups were 56.9% vs. 37.5% (*p*=0.010) (Table 1, 2 and Figure 2).

**Table 2:** Median survival periods and five-year overall survival in recurrent or nonrecurrent RCC cases with or without chemotherapy

	Total cases			Nonrecurrent cases			Recurrent cases			Without chemotherapy cases			Chemotherapy cases		
	No. of patients (%)	Median survival period (months)	Five year OS (%)	No. of patients (%)	Median survival periods (months)	Five year OS (%)	No. of patients (%)	Median survival periods (months)	Five year OS (%)	No. of patients (%)	Median survival periods (months)	Five year OS (%)	No. of patients (%)	Median survival periods (months)	Five year OS (%)
ABCG2^−^	58.7 (84/143)	77.65	72.0	67.7 (65/96)	65.00	71.3	40.4 (19/47)	76.93	69.4	75.6 (31/41)	85.00	96.4	52 (53/102)	75.1	61.0
ABCG2^+^	41.3 (59/143)	51.32	31.2*	32.3 (31/96)	63.70	52.1	59.6 (28/47)	45.41	4.9*	24.4 (10/41)	70.73	77.1	48 (49/102)	48.68	22.6*
OCT-4^−^	65.0 (93/143)	68.11	56.9	38.1 (75/96)	70.92	63.2	38.3 (18/47)	56.67	38.9	75.6 (31/41)	74.16	90.1	60.8 (62/102)	59.43	45.7
OCT-4^+^	35.0 (50/143)	48.92	37.5*	21.9 (21/96)	66.17	61.9	61.7 (29/47)	47.58	11.4*	24.4 (10/41)	65.00	85.7	39.2 (40/102)	47.19	25.1*

^*^ *p*<0.05

## DISCUSSION

This research suggests that ABCG2 and OCT-4 play important roles in the malignant biological behavior of colon cancer, while no correlations of ABCG2 and OCT-4 expression were found with the age, gender, tumor size, or tumor shape of RCC patients. ABCG2 expression significantly correlated with TNM stage, tumor differentiation, and lymphovascular invasion. Additionally, the expression of both ABCG2 and OCT-4 was significantly correlated with RCC recurrence and poor outcomes.

Numerous recent studies have reported poor prognosis and a tendency toward recurrence and metastasis in RCC compared with LCC. The clinical characteristics of RCC cells are similar to the biological features of CSCs. However, few studies have demonstrated a relationship between RCC prognosis and CSCs, particularly a correlation with the expression of CSC markers.

According to the CSC theory, only a small population of cancer cells, called TICs, possesses stem cell potency and is responsible for cancer growth, metastasis and recurrence. To date, CSCs have been isolated using flow cytometry from the tumors of numerous types of cancer, such as breast cancer [14, 15], lung cancer [16–18], liver cancer [19, 20], pancreatic cancer [21], prostate cancer [22], and colorectal cancer [23, 24]. CSCs typically express certain CD molecules identified as CSC markers; however, conflicting results have been reported regarding colorectal CSC markers. For example, according to several studies, CD133^+^ cells possess stem cell potency, and CD133 is a potential CSC marker in colon cancer [25–27]. However, based on other studies, CD133^−^ cells may possess an enhanced capacity to self-renew and differentiate compared with CD133^+^ colon cancer cells [28, 29]. Thus, here, we focused on the widely accepted CSC markers ABCG2 and OCT-4.

ABCG2, also known as breast cancer resistance protein (BCRP), is an ABC family efflux protein that confers resistance to many drugs and radioactivity. ABCG2 has been described as a stem cell marker for side population cells (SP) in several tumors [30–33]. Similar to stem cells, SP cells possess the stem-like characteristics of self-renewal, differentiation, and tumorigenicity [34, 35]. In our previous study, SP cells were isolated from gallbladder carcinoma cell lines [36] and colon cancer cell lines with high ABCG2 expression, and this small population of cells was able to self-renew and differentiate into both SP cells and non-SP cells. These cells also exhibited increased tumorigenicity and drug resistance.

OCT-4, and octomer-binding family protein, is expressed in early embryos and is a key regulator of stem cell pluripotency [37]. Through its transcription factor activity, OCT-4 possesses the ability to maintain cells in an undifferentiated and pluripotent state by maintaining embryonic stem cell self-renewal [38]. Ponti et al. identified breast carcinoma CD44^+^/CD24^−/low^ cells exhibiting stem/progenitor cell properties that highly expressed OCT-4. Moreover, as few as 10^3^ CD44^+^/CD24^−/low^ cells were sufficient to drive tumor formation [39]. As a gatekeeper of embryonic stem cell pluripotency, OCT-4 is also associated with the prognosis of several carcinomas. According to Kosaka et al., high OCT4 expression is an independent prognostic indicator of prostate-specific antigen recurrence [12]. Moreover, OCT-4 overexpression in the absence of SOX2 expression is strongly associated with poor prognosis in cervical cancer [40]. These results are similar to our results.

OCT-4 is rarely expressed in normal tissues. A report showed that ABCG2 may be expressed in the small and large intestine is in contrast to the findings of our study [41]. It is possible that the multidrug resistance protein ABCG2 is only expressed after the activation of quiescent stem cells in normal tissues.

In the present study, ACBG2 and OCT-4 were detected in tissue samples obtained after RCC resections, and the expression of both proteins was significantly correlated with RCC recurrence. These results may be attributable to the fact that ABCG2 belongs to a family of cytomembrane efflux proteins and is involved in the resistance to numerous drugs and radioactivity. Chen et al. also reported an association between ABCG2 overexpression and poor prognosis for hepatocellular carcinoma in elderly patients [42]. However, a different survey found no significant relationship between ABCG2 expression and the clinical outcomes of pediatric sarcomas [43]. ABCG2 may not be enriched in sarcomas, which would explain this result. Another study reported high expression of ABCG2 and OCT-4 in enriched CD90^(+)^CD133^(+)^ liver CSCs and a close association with chemotherapy drug resistance [44]. However, our experiments identified no significant relationship between OCT-4 expression and the clinicopathological variables of RCC. OCT-4 may act as an ON and OFF switch in CSCs. Furthermore, although OCT-4 expression is typically stable, OCT-4 regulation is complex and primarily depends on the microenvironment [45].

In summary, our study describes the relationship between ABCG2 and OCT-4 expression and the clinicopathological characteristics of RCC patients. ABCG2 and OCT-4 expression was significantly correlated with RCC recurrence, which has a poor prognosis. Therefore, ABCG2 and OCT-4 may be indicators of RCC recurrence. Our results may help to inform the mode of treatment for RCC patients and screen for poor outcomes to allow for timely intervention.

## MATERIALS AND METHODS

### Cell culture and transfections

The SW480 cell line was obtained from the Center laboratory of the Tianjin Medical University Cancer Institute and Hospital. SW480 cells were cultured in RPMI-1640 medium supplemented with 10% fetal bovine serum (FBS), L-glutamine, and 1% penicillin/streptomycin at 37°C in a 5% CO_2_ incubator. The ABCG2 and OCT-4 knockdown lentiviruses (sh-ABCG2 and sh-OCT-4) were constructed by and purchased from Genechem (Shanghai, China). All transfections were carried out according to the manufacturer's instructions.

### Xenograft experiments

A total of 1×10^5^ transfected cells were subcutaneously injected into the right armpit of BALB/c nude mice. The weight of the mice and the diameter of tumors were measured every week.

### Spheroid colony-formation assay

The sorted tumor cells were suspended in serum-free DMEM/F12 (1:1 volume, Gibco) supplemented with 20 ng/ml human recombinant EGF (Invitrogen), 20 ng/ml bFGF (Invitrogen) and 5 μg/ml insulin (Sigma), and then cultured in 96-well culture plates. Fresh serum-free DMEM/F12 (described above) was added to each well (0.025 ml/well) every day. After 2–3 weeks, each well was examined using a light microscope, and the total number of spheroid colonies in the 96-well plate was counted. Images of the spheroid colonies were recorded using an inverted microscope (Nikon).

### Flow cytometry and FACS analysis

The cells were resuspended in DMEM with 2% FBS at a concentration of 10^6^/100 μl and incubated for 30 min at room temperature with a 100-fold dilution of the anti-CD133/1-phycoerythrin (eBioscience) antibody. After incubation, the samples were washed twice with PBS/2% FBS and resuspended. Then, 4,6-diamidino-2-phenylindole (1 μg/ml, Sigma) was added to the samples to eliminate the dead cells. Flow cytometry analysis was performed using FACSAria (BD Immunocytometry Systems). The cells were routinely sorted twice and then reanalyzed for purity, which was typically >90%.

### Patients and specimens

The pathology specimens were obtained at Tianjin Medical University Cancer Institute and Hospital between 2004 and 2012 from patients with histopathologically confirmed RCC who underwent radical right hemicolectomy. The clinical characteristics and outcomes were collected until death or loss of follow-up by telephone and mail. In total, 144 cases were followed (1 patient died immediately postoperatively and was excluded from this study), including 47 cases that were recurrent after surgical resection and 96 cases that were not recurrent after the initial surgery. In addition, 102 patients underwent chemotherapy after the operation. The patients’ ages ranged from 21 to 93 years (mean ± standard deviation (SD): 63.1±11.7 years). The survival time was defined as the time between the original operation and the date of death. The Ethics Committee of Tianjin Medical University Cancer Institute and Hospital approved this survey.

### Immunohistochemistry staining

Tumor samples (5-μm sections) were formalin-fixed and paraffin-embedded. The samples were immersed in a 0.3% hydrogen peroxide solution in methanol for 30 min to block endogenous peroxidase. Then, the samples were blocked in a blocking solution containing 10% normal rabbit serum at room temperature for 90 min, followed by phosphate-buffered saline for 15 min. Next, the sections were incubated overnight at 4°C with the following primary antibody dilutions: 1:100 anti-ABCG2 (SANTA CRUZ, Dallas, Texas, USA) and 1:60 anti-OCT-4 (GeneTex, San Antonio, Texas, USA). Finally, the sections were incubated with 3,3′-diaminobenzidine (DAB) substrate solution (eBioscience) for 3 min for color development, counterstained with 0.1% hematoxylin, dehydrated in ethanol and cleared in xylene. The negative controls were obtained by performing all of the above steps except the primary antibody incubation.

### Immunohistochemistry evaluation

A semi-quantitative evaluation system was employed to obtain the staining scores. The staining intensity was classified into four grades: 0, no staining; 1, weak staining; 2, moderate staining; and 3, strong staining. The percentage of stained cells was graded at four levels: 0, no tumor cells; 1, <10% tumor cells; 2, 10-50% tumor cells; and 3, >50% tumor cells. Scores <4 were defined as negative staining, and scores ≥4 were defined as positive staining [13]. Two pathologists who were blinded to the patient prognosis and other clinicopathological variables performed the repeated measurements. The relationships among ABCG2 and OCT-4 expression, clinicopathological features, tumor recurrence and overall survival were analyzed.

### Statistical analysis

Student's t-test and the Chi-square test were used to calculate the significance of the clinicopathological data. Kaplan-Meier analysis was performed to evaluate overall survival. Log-rank tests were used to compare the marker-negative and marker-positive cases. The Cox hazard regression model was employed for the multivariate analysis. All data analyses and graph creation were performed with GraphPad Prism software (Version 7.0), and *p*<0.05 was considered statistically significant.

## Abbreviations

RCC: Right-sided colon cancer
LCC: left-sided colon cancer
CSCs: cancer stem cells
TICs: tumor-initiating cells
ABCG2: ATP-binding cassette sub-family G member 2
POU5F1: POU family of transcription factors, class 5, factor 1
ABC: ATP-binding cassette
FDA: Food and Drug Administration
FBS: fetal bovine serum
